# Single cell sequencing reveals cell populations that predict primary resistance to imatinib in chronic myeloid leukemia

**DOI:** 10.18632/aging.104136

**Published:** 2020-11-23

**Authors:** Weilong Zhang, Beibei Yang, Linqian Weng, Jiangtao Li, Jiefei Bai, Ting Wang, Jingwen Wang, Jin Ye, Hongmei Jing, Yuchen Jiao, Xixi Chen, Hui Liu, Yi-Xin Zeng

**Affiliations:** 1 Department of Hematology, Lymphoma Research Center, Peking University Third Hospital, Beijing 100191, China; 2 State Key Lab of Molecular Oncology, National Cancer Center/National Clinical Research Center for Cancer/Cancer Hospital, Chinese Academy of Medical Sciences and Peking Union Medical College, Beijing 100021, China; 3 Department of Hematology, Beijing Hospital, National Center of Gerontology, Beijing, China; 4 Department of Hematology, Beijing Tongren Hospital, Capital Medical University, Beijing, China; 5 Genetron Health (Beijing) Co. Ltd., Beijing 102206, China; 6 Children’s Hospital of Chongqing Medical University, Chongqing 400014, China; 7 Department of Experimental Research, Sun Yat-sen University Cancer Center, State Key Laboratory of Oncology in Southern China, Collaborative Innovation Center for Cancer Medicine, Guangzhou, Guangdong Province, China

**Keywords:** chronic myeloid leukemia, peripheral immune structure, single cell sequencing, TKI resistance, stem cells

## Abstract

The treatment of chronic myeloid leukemia (CML), a disease caused by t(9;22)(q34;q11) reciprocal translocation, has advanced largely through the use of targeted tyrosine kinase inhibitors (TKIs). To identify molecular differences that might distinguish TKI responders from non-responders, we performed single cell RNA sequencing on cells (n = 41,723 cells) obtained from the peripheral blood of four CML patients at different stages of treatment to generate single cell expression profiles. Analysis of our single cell expression profiles in conjunction with those previously obtained from the bone marrow of additional CML patients and healthy donors (total = 69,263 cells) demonstrated that imatinib treatment significantly altered leukocyte population compositions in both responders and non-responders, and affected the expression profiles of multiple cell populations, including non-neoplastic cell types. Notably, in imatinib poor-responders, patient-specific pre-treatment unique stem/progenitor cells became enriched in peripheral blood compared to the responders. These results indicate that resistance to TKIs might be intrinsic in some CML patients rather than acquired, and that non-neoplastic immune cell types may also play vital roles in dispersing the responsiveness of patients to TKIs. Furthermore, these results demonstrated the potential utility of peripheral blood as a diagnostic tool in the TKI sensitivity of CML patients.

## INTRODUCTION

Chronic myeloid leukemia (CML) is a hematopoietic stem cell disease caused by a single translocation event, t(9;22)(q34;q11), which generates the fusion protein BCR-ABL. CML responds to treatment with targeted tyrosine kinase inhibitors (TKI), such as imatinib and dasatinib, which bind to the active site of BCR-ABL. However, some CML patients fail to benefit from TKI therapy, and 3-5% progress into blast crisis after treatment [1–3]. Underlying mechanisms for resistance to TKI treatment have been associated with genomic aberrations in addition to the BCR-ABL translocation, including mutations in the fusion protein and triosomy of chr8 or chr17p [4, 5].

Immune function in CML is impaired as in other malignancies. For example, Rossignol et al. [6] reported dysfunctional invariant natural killer T cells (iNKT) in untreated CML patients, and Chen et al. [7] also observed a decreased proportion of NK cells in the peripheral blood that did not recover after treatment with TKIs. In addition, immune suppressive cells, such as Tregs and myeloid-derived suppressor cells (MDSCs), were also reported to increase in high-risk populations [8]. Despite these intriguing results, the population structure of the peripheral blood has not been well characterized in CML patients. The extent to which the population structure of the peripheral blood differs among patients, whether these differences have an impact on the response to TKI treatment and how the TKI treatment alters the immune system have also not yet been fully elucidated. In fact, an immune-modulatory role for TKI treatment has been previously observed in studies examining the frequencies of different immune compartments in the peripheral blood [9]. Yet, the specific changes in the expression programs remain a mystery.

Recent advances in single cell analysis have challenged and revised the trajectory map of hematopoiesis [10–12]. Thus, to refine the immune cell population structure at a higher resolution in CML might lead to further insight into alternative therapies. Giustacchini et al. and Warfvinge et al. [13, 14] analyzed single-cell transcriptomes of CML cancer stem cells in bone marrow and identified subpopulations and gene markers of TKI therapy-resistance. However, there are some limitations of these studies. First, these studies examined only a subgroup of CD34+ cells rather than whole CML cell populations. Second, only a small number of cells were studied (~ 2,000 cells), which limited cell classification. Finally, cells were only characterized from the bone marrow, and not the peripheral blood, which can be used as a noninvasive biopsy for monitoring of CML. Here, we performed single cell RNA-sequencing on a total of 41,723 cells from peripheral blood, covering different TKI treatment stages of four CML patients, to generate expression profiles for individual cells. We furthermore analyzed this data in association with expression profiles of 17,540 bone marrow cells from both CML patients and healthy donors to generate a comprehensive landscape for CML. Using the combined datasets, we investigated the immune cell structure in parallel with CML progression, and characterized the molecular/cell signature of the immune response to TKI treatment. Our study provides insight into the pathogenic mechanisms involved in CML beyond BCR-ABL translocation and new therapeutic strategies to complement TKI.

## RESULTS

### Comparison of the expression profiles of primitive stem/progenitor populations in peripheral blood from CML patients with healthy bone marrow components

To investigate the comprehensive immune cell composition in the peripheral blood of CML patients, we performed single cell RNA sequencing (scRNA-seq) on PBMCs collected from four representative patients with CML at multiple time points before (BT) and after treatment with imatinib. Despite treatment with imatinib, one patient (P04) rapidly progressed to the blast phase within three months, and had acquired a mutation in the kinase domain region of BCR-ABL (p.M448V). This patient received dasatinib for the second round of treatment. PBMC samples from P04 in the blast phase as well as post-blast treatment (blast cells (BC)-BT and BC-AT, respectively) were also included. We also obtained PBMCs from a single healthy donor (N). These single-cell suspensions were subjected to scRNA-seq using barcodes and UMIs for individual cells and unique transcript counting. After filtering out low-quality cells, we obtained a total of 41,723 cells from peripheral blood with at least 200 detected genes (Supplementary Table 2). The cell numbers obtained from different stages including all individuals were the following: normal, 5,082; BT, 5,562; AT, 24,815; BC-BT, 4,515; and BC-AT, 1,749.

**Figure 1:**
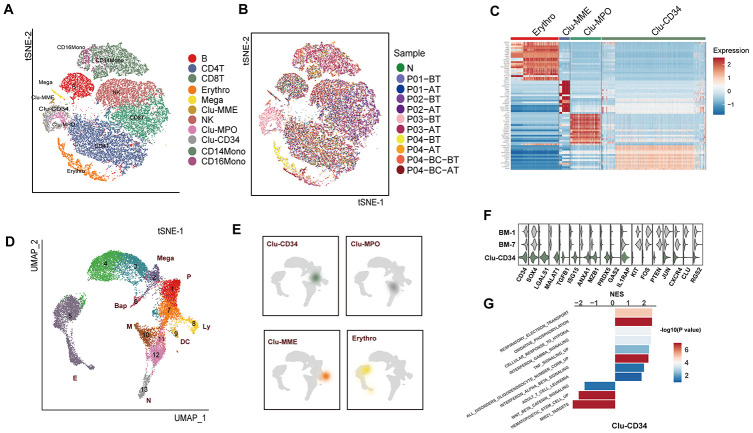
**Comparison of the primitive stem/progenitor populations in peripheral blood with the healthy BM components.** (**A**) TSNE plot for the resultant 11 clusters identified based on single cell RNA sequencing. The total number of cells is 41,723. Clusters are highlighted by different colors, and the number of cells in each cluster is listed in Supplementary Table 2. (**B**) TSNE plot of all cells. Colors indicate sample origin. (**C**) Heatmap showing the expression profiles of the four primitive clusters identified in peripheral blood. Expression of the scaled value of the top 30 significant marker genes in each cluster are shown. (**D**) UMAP plot displaying the resultant clusters identified in healthy Lin- bone marrow datasets [15]. The total number of cells is 15,253. Colors indicate clusters, and lineage destinations are labeled. P, early progenitor cells; Meg, megakaryocytes; (**E**) erythroid cells; BaP, basophil progenitors; N, neutrophils; M, monocytes; DC, dendritic cells; Ly, lymphoid cells. (**E**) Kernel density plot showing the projection result of the four primitive clusters identified in peripheral blood onto the BM reference map. (**F**) Violin plot showing the expression distribution of selected genes in Clu-CD34 in comparison with BM-1 and BM-7. (**G**) Bar plot displaying the GSEA result on the ordered expression profiles in Clu-CD34. X-axis indicates the normalized enrichment score (NES) and colors indicate the -log10(*P* value).

Unsupervised clustering of the expression data obtained from all cells revealed a total of 11 clusters (Figure 1A). Through examination of known cell type specific markers, eight clusters were classified as functionally common immune cell types, including CD4+ T cells, CD8+ T cells, NK cells, B cells, CD14+ monocytes, CD16+ monocytes, and megakaryocytes (Supplementary Figure 1A). Sample-of-origin of cells in these immune cell compartments were well mixed (Figure 1B, Supplementary Figure 1B), and the fractions of each compartment in the healthy blood sample were consistent with the previously reported composition of PBMCs, thus confirming the clustering methods and results (Supplementary Figure 1C).

The most remarkable result was the identification of four clusters with apparent features of primitive cells, including Clu-CD34, Clu-MPO, Clu-MME and a subset of erythrocytes (GATA1^high^) (Figure 1C). These four clusters exhibited a significant enrichment in the patients with poor prognosis, such as Clu-CD34 and Clu-MPO in P03 at diagnosis, early-stage erythrocytes (GATA1^high^) in P03 and P04 at diagnosis, and Clu-MME in P04 at the blast-crisis stage (Figure 1B, Supplementary Figure 1D. To further understand the biological status of these primitive cells, we used an expression dataset representing healthy Lin- bone marrow cells (n=17,540) as a comprehensive reference (referred to as BM-reference) [15]. Visualization using uniform manifold approximation and projection (UMAP) effectively recapitulated the intermediate clusters from our analysis during the continuous development process (Figure 1D, BM-1 to BM-11). We then mapped the primitive cells from peripheral blood onto the BM-reference to understand the hierarchy of these cells (Figure 1E). Clu-CD34 correlated with a collection of early stem cells with heterogenous differentiation destinies. The blended lineage potentials in this cluster were also confirmed using the lineage-specific signatures defined in a separate study (Supplementary Figure 1E). The primitive cell cluster Clu-MPO contained cells from the earliest myeloid progenitors to neutrophil-defined or monocyte-defined progenitors, while the Clu-MME cluster represented early B cell progenitors.

Since BCR-ABL fusion is usually considered to be associated with CD34+CD38- stem cells, we focused on the primitive cell cluster Clu-CD34 and compared the expression profile of this cluster with early HSCs and their immediate progenies from the reference dataset (BM-1 and BM-7) to identify differentially expressed genes (DEG). Up-regulated DEGs included *LGALS1*, *MALAT1*, *TGFB1*, *MZB1*, while down-regulated DEGS included *KIT*, *PTEN*, and *CXCR4* (Figure 1F). GSEA analysis revealed that inflammation signatures (interferon signaling, TNF signaling) were significantly up-regulated in Clu-CD34 which was consistent with an enhanced inflammatory response in these patients (Figure 1G). These same inflammation signatures were also associated with Clu-MPO (Supplementary Figure 1F). Clu-CD34 and Clu-MPO were mainly composed of cells from the non-responder P03 at diagnosis. Most of the altered expression signatures identified in these two clusters were consistent with TKI non-responding signatures identified in previous single cell studies performed on bone marrow samples from CML patients. For example, Giustacchini et al. [13]. observed enrichment of signatures related to inflammation, TGF-beta and TNF-alpha in BCR-ABL**-** stem cells at diagnosis from poor relative to good responders. In addition, a subgroup of BCR-ABL+ stem cells with selective persistence during TKI treatment was found exhibiting increased expression of *TGFB1*, *GAS2*, *CTNNB1*, and *HIF1A* but reduced expression of *CXCR4*, which was consistent with our observations. Finally, TNF-alpha and TGF-beta signaling became progressively enriched in this subgroup during the course of treatment. Warfvinge et al. [14]. also found that the most TKI-insensitive cells were negative for cKIT. Results from all the datasets suggested that despite the extensive heterogeneity that was present among CML patients, some of the features were shared by TKI-insensitive cells in the bone marrow, and these features could also be detected in the peripheral blood of some patients.

### Integration analysis of bone marrow and peripheral blood primitive cells in CML patients

**Figure 2:**
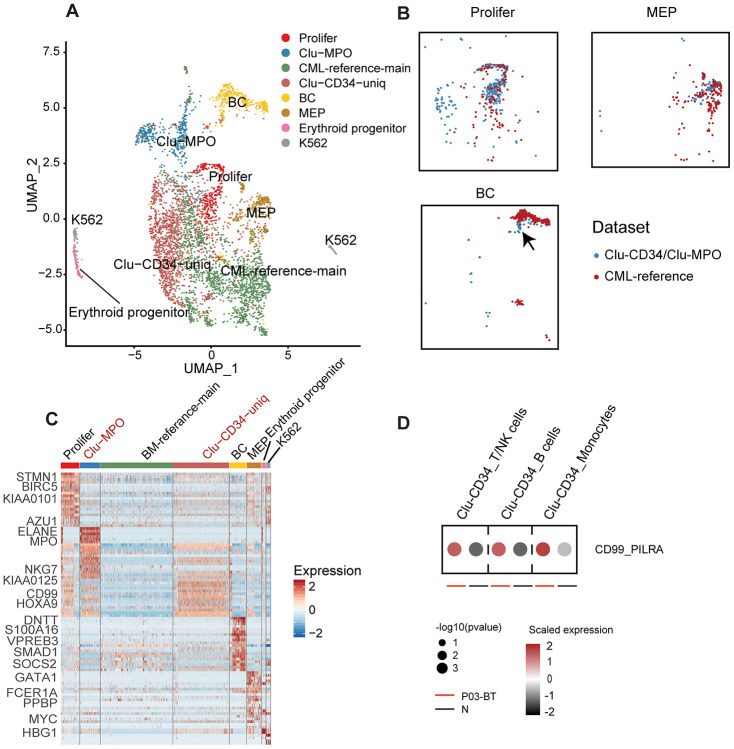
**Comparison of Clu-CD34 and Clu-MPO with bone marrow CML datasets.** (**A**) UMAP plot showing the clustering result of the integrated datasets comprising Clu-CD34, Clu-MPO and the BM-reference [13]. The total number of cells is 4,603 (Clu-CD34: 1,789; Clu-MPO: 527; BM-reference: 2,287). (**B**) UMAP plots of cells. Colors indicate dataset origin. Arrow in the lowest panel indicates the mixture of cells from the Clu-CD34 (n = 61) clustered together with BC cells from the BM-reference. (**C**) Heatmap showing expression of marker genes across different subtypes from the integrated dataset. Two specific subtypes from our dataset (absent in the BM-reference) are marked as red at top. (**D**) Dot plots comparing the interaction of the CD99-PILRA ligand-receptor pair between P03-BT and N. The p value was calculated using a permutation test.

We next combined the primitive clusters in peripheral blood and the stem cells in bone marrow (CML-reference) [13] to illustrate the variation from BM to peripheral blood in CML patients. The CML-reference contained 2,287 CD34+CD38- cells from 34 CML patients and 6 healthy controls. Integration of Clu-CD34 and Clu-MPO with the CML-reference revealed both well-mapped and unmapped cells (Figure 2A). Using specific cell type markers, we identified that the well-mapped cells included three groups of cells: proliferating, megakaryocyte/erythrocyte progenitors (MEP) and erythroid progenitors (clustered together with K562 cells) (Figure 2B). Interestingly, we also found that a small subset of the cells in Clu-CD34 grouped together with the cells at the blast crisis stage within the CML-reference (Figure 2B). This observation may provide evidence for the existence of CML stem cells at diagnosis which already have potential for seeding blast crisis. However, two groups of cells clustered separately from the CML-reference, including a subset of Clu-CD34 cells and most Clu-MPO cells. The unmapped subset in Clu-CD34 uniquely expressed several leukemia-related genes (Figure 2C), one of which was *CD99*. This gene is known for playing key roles in promoting the mobilization of the hematopoietic cells [16–18] and has been recognized as a robust marker and promising therapeutic target in several tumor types [19]. Through inspection of known ligand-receptor pairs, we found that Clu-CD34 could possibly affect other immune cell types through elevated interaction between CD99 and PILRA (Figure 2D). Finally, *HOXA9*, which is frequently overexpressed in acute myeloid leukemia and may promote leukemia through epigenetic landscape remodeling [20, 21], was up-regulated.

**Figure 3:**
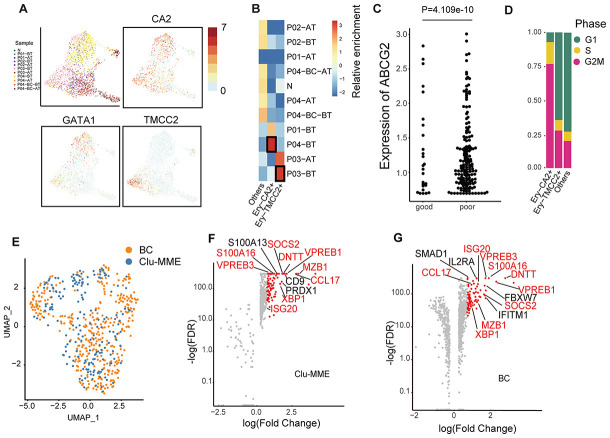
**Comparison of erythrocytes and Clu-MME with bone marrow CML datasets.** (**A**) UMAP plot presenting the re-clustering result of erythrocytes. Cells are highlighted with colors indicating sample origin. The expression of *GATA1*, *CA2* and *TMCC2* are indicated. The *CA2*- and *TMCC2*-expressing erythrocytes are separately enriched in P04-BT and P03-BT. (**B**) Heatmap showing enrichment of samples in each erythroid cluster. Enrichment score was calculated using the Fisher’s exact test and indicated by log10(Odd Ratio). (**C**) Beeswarm plots showing the expression of *ABCG2* between good responders (P01 and P02) and poor responders (P03 and P04). P= 4.109e-10, unpaired t test. (**D**) Bar plots showing the fraction of cells from different cell cycle phases across different erythrocyte subtypes. (**E**) UMAP plot showing the integration result of the BC cluster (from the integrated dataset shown in Fig. 2a) and Clu-MME (the numbers of cells in the BC cluster and Clu-MME are 370 and 183, respectively). (**F**) Scatter plot showing the highly-expressed marker genes in Clu-MME (left) and the BC cluster (right). Significant markers (FDR<0.05, fold change > 2) are shown as red dots. The name of shared marker genes of these two clusters are indicated in red. (**G**) Heatmap comparing the expression profiles of CD16+ monocytes across different samples. The selected marker genes are indicated.

Erythropoiesis is one of the major hallmarks of CML. In the peripheral blood dataset, we did observe a large number of erythroid cells with extremely high expression of *HBA1/2* and *HBB*, although most of these cells were filtered out due to the low number of detected genes. However, among all the erythroid cell types, the most primitive erythroid progenitors (GATA1^high^) originated overwhelmingly in the non-responder P03 and the fast-BC-transforming patient P04 at diagnosis (Ery-TMCC2+ from P03-BT and Ery-CA2+ from P04-BT, Figure 3A, 3B). Compared with the GATA1^low^ erythroid cells, these GATA1^high^ cells had higher expression of *ABCG2*, which is an ATP-binding cassette (ABC) transporters and has been implicated as a potential mechanism of primary resistance to TKI (Figure 3C) [22]. Interestingly, cells in the Ery-CA2+ cluster (originated from P04) were predominantly synchronized in the G2/M phase (Figure 3D). In previous studies, cells just entering terminal differentiation have been identified in committed erythroid progenitor initiates, in part due to their synchronization in S phase [23, 24]. This cluster therefore possibly represents erythroid-terminal differentiation initiating populations.

In the bone marrow dataset, cells from the BC phase were separated from those from the chronic phase. In the peripheral blood dataset, we also found that Clu-MME, composed of 184 cells, was significantly enriched for blast phase cells from P04 (Supplementary Figure 1D, Figure 2A). This group of cells expressed common acute lymphocytic leukemia antigen *MME*, and the pro-B cell specific gene *VPREB1*, indicating that a minor subpopulation of P04-BC-BT was a lymphocytic cell type. We then integrated the BC cells from bone marrow and peripheral blood, and observed a well-mixed cell population [13] (Figure 3E). This result indicated that cells at the BC phase had unique expression signatures, and probably exhibited less inter-patient heterogeneity. The exclusively up-regulated genes in BC cells included *SOCS2* and *S100A16*, which are pivotal in promoting progression of leukemia as well as other types of cancer (Figure 3F, 3G) [25–27]. In addition, Clu-MME from peripheral blood also exhibited signatures consistent with a *RUNX1*-*RUNXT1* translocation, indicating that selection for an additional genomic alteration had occurred in P04, even though it was represented by only a small fraction of cells (Supplementary Figure 2B). This detection of this translocation in this small cell population also emphasizes the high sensitivity of single cell transcriptome data in the identification of subclonal genetic alterations.

### Validation of the pre-treatment predictive signatures

**Figure 4:**
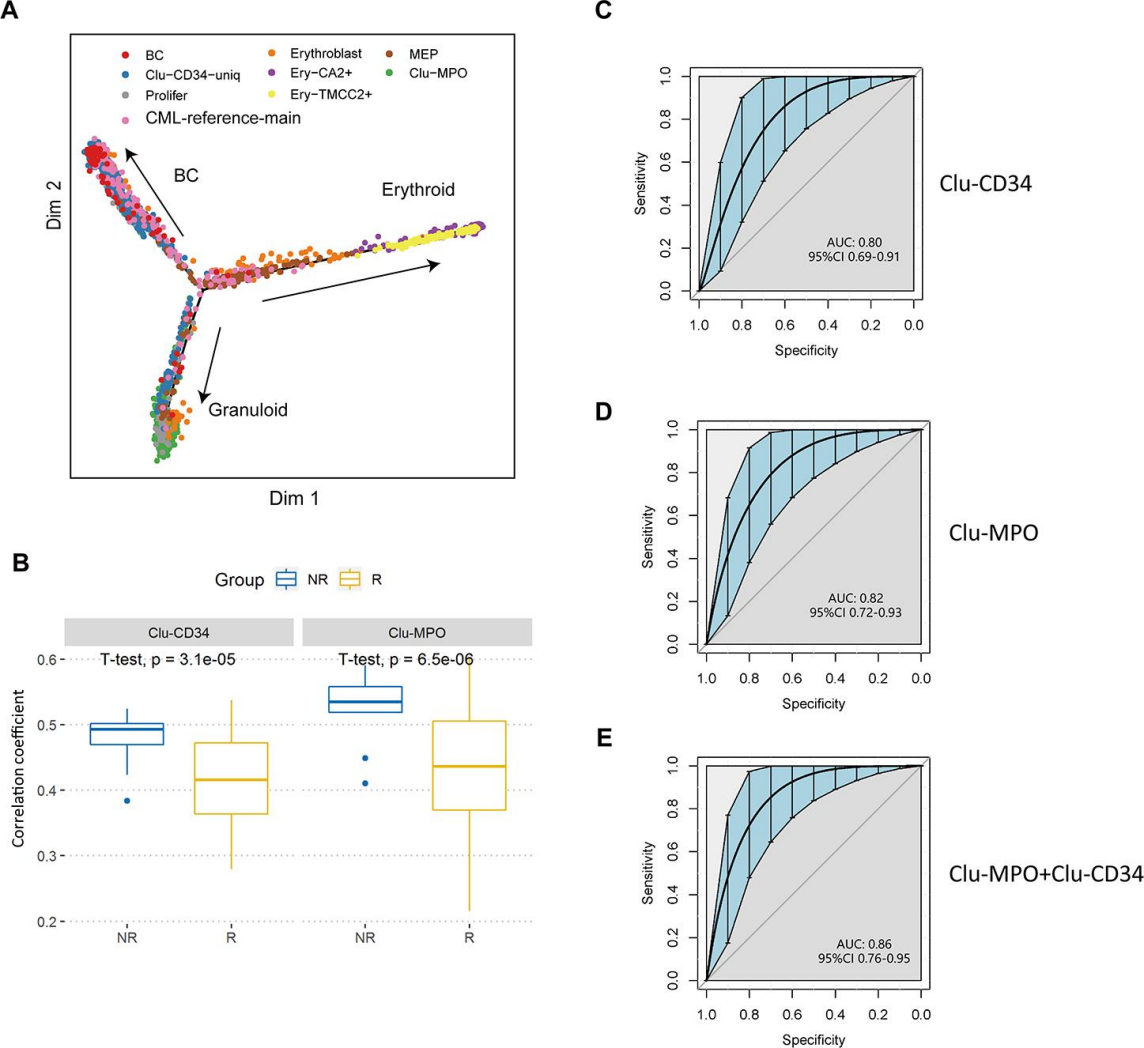
**Validation of prognostic cell populations in 59 CML patients treated with imatinib.** (**A**) Pseudotime trajectory of the primitive cells in peripheral blood. Cells are colored based on their identities in the integrated datasets with CML-reference or the erythroid clusters. (**B**) Box plots comparing the correlation coefficients of Clu-CD34 (left) and Clu-MPO (right) between 13 imatinib nonresponders (NR) and 83 responders (R) from GSE130404. The correlation coefficients were calculated using Pearson correlation of gene expression signatures of these two clusters with the gene expression profile in each CML patient treated with imatinib (see Methods). (**C**, **D**) ROC curves illustrating the classification performance of Clu-CD34 (c) and Clu-MPO (d) gene expression signatures of 13 imatinib nonresponders and 83 responders from GSE130404. The blue shade denotes the 95% confidence interval of the sensitivity at a given specificity point. AUC, area under the ROC curve are indicated. e) We integrated Clu-CD34 and Clu-MPO clusters together to predict imatinib resistance. ROC curves illustrating the classification performance of gene expression signatures of 13 imatinib nonresponders and 83 responders from GSE130404. The blue shade denotes the 95% confidence interval of the sensitivity at a given specificity point. AUC, area under the ROC curve are indicated.

Clu-CD34, Clu-MPO, Ery-TMCC2, and Ery-CA2 were present in the peripheral blood of patients with a poor-prognosis at diagnosis. These cells showed limited developmental trajectories towards three directions, erythroid, myeloid and BC (Figure 4A). Since they showed unique expression signatures compared with either normal stem cells or peripheral blood cells, we assumed that these primitive clusters could possibly predict the prognosis of CML patients as early as at primary diagnosis. Therefore, we analyzed a transcriptome dataset of 59-patients with known imatinib-treatment response [28], and found that the expression signatures of Clu-CD34 and Clu-MPO had significantly higher correlations with the imatinib nonresponders compared with the imatinib responders, (Supplementary Figure 2C; Clu-CD34, P=0.0024, Clu-MPO, P=0.0068; unpaired t test). We generated ROC curves to demonstrate the prediction performance of Clu-CD34 and Clu-MPO gene expression signatures on the dataset. The curves were able to predict resistance to imatinib before initiation of the therapy (Supplementary Figure 2D; Clu-CD34, AUC = 0.74, Clu-MPO, AUC = 0.69).

We analyzed three other independent datasets derived from cohorts of patients (total = 217) treated with imatinib or dasatinib, or in different phases of CML, to achieve a significant scientific conclusion [29–31]. For the CML patients treated with imatinib (n = 96, GSE130404), Clu-CD34 and Clu-MPO gene expression signatures were able to predict early resistance to imatinib (Clu-CD34, AUC = 0.80, Clu-MPO, AUC = 0.82; Figure 4B–4D). When we integrated these two clusters together, the AUC reached 0.86 (95%CI 0.76-0.95; Figure 4E). However, in patients treated with dasatinib (n = 14, GSE33224), Clu-CD34 and Clu-MPO gene expression signatures were not able to predict dasatinib resistance (P-value > 0.05; Supplementary Figure 2E, 2F). These two clusters therefore are of more value in predicting response to imatinib than dasatinib. In the last cohort of CML patients in different phases of the disease (n = 107, GSE4170), Clu-CD34 and Clu-MPO gene expression signatures were highly associated with blast crisis phase (P < 0.05; Supplementary Figure 2G, 2H).

### Modulation of the immune structure in response to imatinib treatment

**Figure 5:**
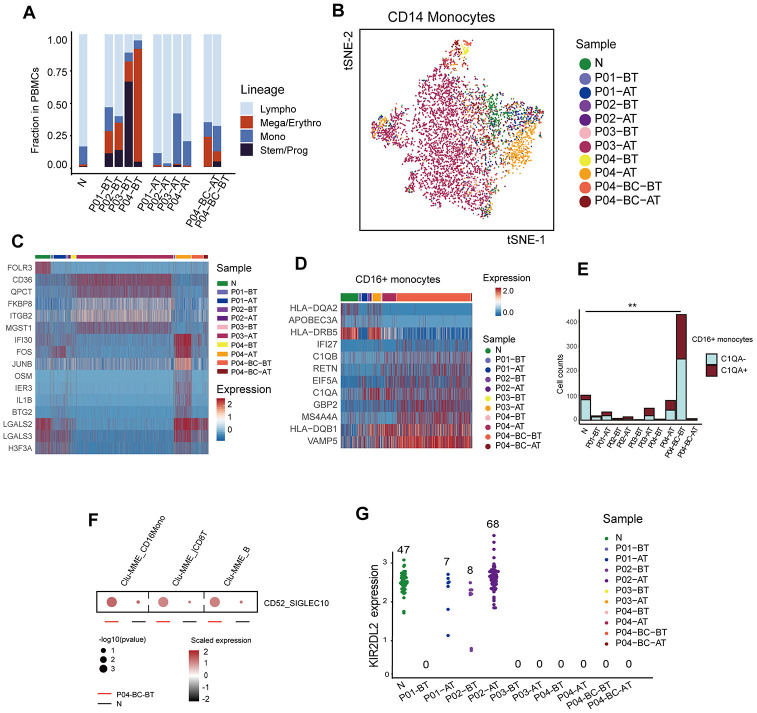
****Modulation of the immune structure in response to imatinib treatment (**A**) Bar plots showing the fraction of different cell lineages in each sample. (**B**) TSNE plot of the re-clustering result of CD14+ monocytes. Cells are highlighted in colors indicating sample of origin. The number of cells in each cluster is listed in Supplementary Table 2. (**C**) Heatmap displaying the expression profiles of CD14+ monocytes across different samples. Top differentially expressed genes in each sample are indicated. (**D**) Heatmap comparing the expression profiles of CD16+ monocytes across different samples. The selected marker genes are indicated. (**E**) Bar plots comparing the detected *C1QA*-expressing monocytes across different samples. P value was calculated using a Fisher’s exact test. (**F**) Dot plots comparing the CD52-SIGLECT10 ligand-receptor interactions between P04-BC-BT and N. The p value was calculated using a permutation test. (**G**) Beeswarm plots showing the expression of *KIR2DL2* among all cells across samples. The number of cells expressing *KIR2DL2* in each sample is indicated.

In addition to the primitive cell populations, we investigated changes in the common functional non-neoplastic immune compartments during the treatment course. Fractions of these immune compartments differed significantly among different time points or different response status to imatinib. Within untreated samples (BT), the frequencies of lymphocytes and monocytes were significantly reduced relative to the healthy donor, especially in patients with adverse prognosis (P < 2e-16, Fisher’s exact test), which were dominated by stem/progenitor cells (P03) or erythroid cells (P04). Imatinib treatment had an obvious positive effect in the restoration of the functional immune structures (Figure 5A). However, the monocytes in the poor responders clustered independently from those in healthy donors (Figure 5B, 5C). GSVA analysis revealed aberrant activation of multiple pathways in P03-AT, including the TNF-α signaling, MAPK, and IL12-STAT4 pathways (Supplementary Figure 3A). Moreover, monocytes from P04-AT showed significantly higher expression levels of several genes, including AP-1 members *FOS* and *JUNB* (Supplementary Figure 3B).

We also observed that CD16+ monocytes were significantly enriched with cells originating from P04-BC-BT. They accounted for 49.7% (429/863) of the total number of monocytes within the sample, which was much higher than in the healthy control (101/690, 14.6%; P < 2.0e-16, Fisher’s exact test). Their expression profile was also distinct from CD14+ monocytes (Supplementary Figure 3C, 3D). Several genes, including *IFI27* and *VAMP5*, were highly up-regulated in P04-BC-BT compared to the CD16+ monocytes from the healthy donor (Figure 5D). The increased frequency of macrophages may indicate that there is a pro-inflammatory stimulation in the peripheral blood during the blast crisis phase. In addition, further dissection of CD16+ monocytes revealed a higher proportion of cells expressing *C1QA*, which has been implicated in suppression of the cytotoxicity of CD8+ T cells (Figure 5E) [32, 33]. The Clu-MME in P04-BC-BT also showed evidence for significantly elevated interaction between *CD52* and *SIGLEC10*, which has been shown to suppress T cell function (Figure 5F) [34]. *LAG3* was also increased in P04-BC-BT T cells (Supplementary Figure 3E), revealing suppressed T cell function through the presence of both T cell inhibitory signals and suppressive macrophages during blast crisis.

In addition to the possible interaction between macrophages and T cells, we noticed that the NK cell inhibitory receptor and licensing mediator *KIR2DL2* was only restored in the patients with favorable prognosis after treatment (Figure 5G). Previous studies have suggested that *KIR2DL2* plays a protective role in CML as well as in solid tumor types, representing a good prognostic factor [35, 36]. Expression of NK cell inhibitory receptors are highly heterogeneous among human populations; thus, it was unclear whether this discrepancy in *KIR2DL2* was due to differences in tumorigenesis or in genetic backgrounds among individuals. However, we did not find discrepancies for other inhibitory receptors, including *KIR2DL3*, which is considered to bind *HLA-C1* like *KIR2DL2*, but with lower affinity (Supplementary Figure 3F) [37]. Previous studies have also suggested that KIR polymorphism was associated with clinical response [38]. Although this result requires further validation due to the low number of cells detected with expression of *KIRD2L2*, it might indicate that this gene is crucial in sensitizing NK cells against leukemic cells.

## DISCUSSION

Ultra-high-throughput single cell RNA-seq enabled characterization of the expression profiles of leukemic cells and immune structures simultaneously. Although the number of cases was limited, we managed to identify features of leukemic cells common among patients, and to excavate significant discriminations between poor and favorable prognosis, based principally on the cell population configurations. The presence of even small cell populations with unique expression profiles could be detected. Cells with similar transcriptomes existing in all patients represented “classic leukemic” cells in CML. Thus, molecular analysis at the single cell level has provided a comprehensive overview of many cell populations in peripheral blood of patients, which has implications for management of patients and their response to the current standard of care for CML.

Many of the previously reported genes playing important roles in CML progression or resistance were detected in our study, such as *c-FOS* and *DUSP1* in CD34+ cells [39]. However, preservation of these genes might vary in different subpopulations of terminally differentiated cells, as *c-FOS* was enriched in a subgroup of CD14+ monocytes (Supplementary Figure 3B) and *DUSP1* was elevated in CD16+ macrophages (Supplementary Figure 3G), which were significantly expanded in the BC phase. This observation emphasizes the necessity of understanding the precise role of different lineage priming processes and the exact function of these terminal immune compartments in the context of leukemia.

The presence of sample-specific subpopulations in untreated cells from patients with a poor prognosis indicated that the clinical resistance phenotype is more intrinsic than acquired. Nevertheless, the exact cell subpopulations indicative of clinical TKI resistance varied among patients, and they represented/covered different developmental stages along the trajectory of hematopoiesis. Using a recently revised hematopoiesis map at the resolution level of single cells, we refined the (accurate) hierarchy of the circulating progenitor cells previously determined as common myeloid progenitors (CMP) and megakaryocyte/erythrocyte progenitors (MEP) [40, 41]. The differentiation destiny of the leukemic HSCs in CML was basically confined to granulocytes and erythrocytes, but it may vary among patients. Notably, in the non-responder for imatinib, an accumulation of cells in the population occurred at a higher level in the hierarchy of the differentiation of stem cells, suggesting that restoring the ability of cells to differentiate may further facilitate TKI treatment.

The mutations accompanying the development of cancer continue to be characterized for the purposes of understanding the biology of the disease and the design of therapies specific for malignant cells in individual patients [42–44]. While targeting mutations works for many cancers [45–47], the approach is of significant benefit to CML patients due to the application of TKI targeted at BCR-ABL kinase [48]. However, a significant proportion of CML patients still suffer from the resistance to TKI treatment. Thus, although the prediction of primary resistance of CML patients to imatinib is difficult, it is a clinically important goal to achieve [28].

The uniquely expressed gene sets in resistant-relevant cell populations discriminate them from the normal functional compartments, as well as classic leukemic cells, providing possible surrogates for their identification in clinical application. In our study, we identified two unique cell populations as primary imatinib resistance clusters, which can predict imatinib response before treatment using available CML expression profiles. Of more clinical importance is that these two clusters can be detected in peripheral blood, but not necessarily in bone marrow. Nevertheless, some of the possible prognostic populations were too small, such as the Clu-CD34 subset seeding blast crisis and Clu-MME, to be detected using traditional strategies. These results further highlight the importance and necessity of high-throughput methodologies, such as single cell RNA sequencing.

In addition, molecular distinctions between favorable and poor prognosis was found not only in leukemic stem/progenitor clones, but also in the terminal immune context which consist mainly of macrophage/monocytes and T cells. The observed molecular differences between leukemic monocytes emphasizes the fact that although the morphology might be normal, expression can differ in leukemic myeloid cells. Both macrophages and T cells are known to play vital roles in the suppression of malignancy, which underscores the value of the exploration of immunotherapy in combination with TKI treatment, especially for patients at high risk for resistance or relapse [49].

## MATERIALS AND METHODS

### Ethics statement

The protocols in this study were approved by the Institutional Review Board (IRB) of Beijing Hospital following the guidelines issued by the Ministry of Science and Technology of the People’s Republic of China. Written informed consent was obtained from all individuals participating in the study in accordance with the Declaration of Helsinki. Human samples were collected and anonymously coded.

### Patients and samples

Our study included four newly diagnosed CML patients who had undergone treatment with imatinib between 2017 and 2018 at the Department of Hematology, Beijing Hospital and the Department of Hematology, Beijing Tongren Hospital. One healthy donor was also included in our study. All patients were diagnosed according to the 2008 World Health Organization (WHO) consensus criteria. Peripheral blood mononuclear cells (PBMCs) for single cell RNA sequencing were isolated from 10 mL of peripheral blood within 2 h of the draw using Human Lymphocyte Separation Medium (DAKEWE; Shenzhen, China) according to the manufacturer's instructions. The clinical information is summarized in Supplementary Table 1**.**

### 10X Genomics Single-Cell RNA Sequencing

PBMCs were suspended and loaded on the Chromium Single Cell Controller, and single-cell RNA-seq libraries were prepared following the manufacturer’s instructions using the Single Cell 3’ Library Gel Bead Kit V1 (10 X Genomics; San Francisco, CA, USA). The captured libraries were sequenced on the Illumina HiSeq genome analyzer with paired-end 150-base reads (Illumina; San Diego, CA, USA).

### Data Analysis

### Pre-processing of sequencing data

The sequencing raw data was first de-multiplexed, aligned to the reference genome (hg19, UCSC), and quality-filtered, and barcodes and unique molecular identifiers (UMIs) were counted using CellRanger for each PBMC sample, according to the manufacturer’s instructions. We applied Seurat v2.3.4 [50] and Seurat v3.5.2 [51] for merging of samples, filtering (cells with > 200 genes detected, genes with > 3 cells detected, and cells with > 5% UMIs derived from mitochondrial genes), down-sampling, data normalization and scaling.

### Integration of datasets

Integration of datasets from different studies was generated with procedures implemented in Seurat [51]. In brief, features with high variances in each dataset were first selected individually, and those features identified as highly-variable in multiple datasets were combined. The top ranked 1,000 to 2,000 features were selected for downstream processing. The paired datasets were then placed in sharing low-dimension spaces using canonical correlation analysis (CCA). Mutual nearest neighbors (MNN) were then identified based on K-nearest neighbors (KNN), and these MNNs were then defined as “anchors” connecting each pair of datasets. The parameter *K.filters* in the command *FindIntegratedAnchors* was set to 50 when dealing with small sized datasets with < 500 cells. Datasets were then assembled using the identified anchors and processed as a single scRNA-seq object for subsequent analysis.

### Dimension reduction and unsupervised clustering of the data

Principle component analysis (PCA) was performed using variable genes with *RunPCA* implemented in Seurat. The standard deviations of the first 40 principle components (PCs) were plotted to determine which PC would be used for further clustering and dimension reduction. Unsupervised clustering was performed using *FindClusters* in Seurat. Clustering results were visualized in 2-dimensional images by applying t-distributed Stochastic Neighbor Embedding (t-SNE) or Uniform Manifold Approximation and Projection (UMAP) [52]. Cluster-specific markers and differentially expressed genes among clusters or between any two given cell groups were identified using *FindAllMarkers* or *FindMarkers* in Seurat, and cell types were determined using the known lineage-specific markers listed in Supplementary Figure 1A. The scores of different features were calculated based on the average expression levels of the feature-related gene sets (Supplementary Table 3) using *AddModuleScore* in Seurat. Cell cycle status was imputed with *CellCycleScoring* in Seurat, using implemented *cc.genes* for scoring and phase mapping (Supplementary Table 3).

### Projection of single cells onto reference maps

When projecting the query dataset onto the reference dataset, we first selected 2,000 of the most variable genes among the intersecting genes between the reference and query datasets. Using these 2000 features, we calculated the PCs in the reference dataset, and the top 30 PCs were projected onto the query dataset. For each cell from the query dataset in the common lower dimensional space, the nearest five cells from the reference dataset were considered as the anchors of the query cell. The projection result is represented by a kernel density plot of the anchors on the reference map.

### Pathway and gene set analysis

Standard GSEA [53] or GSVA [54] was applied for the identification of significantly enriched gene sets between two clusters or given cell groups. The required input files (including *.gct and *.cls) were extracted from the expression matrix (the integrated transformed matrix was used for integrated datasets), and the gene set files were downloaded from The Molecular Signature Database (MSigDB) [55]. Hallmark, curated and GO gene sets (H, C2 and C5, respectively) from MSigDB were used for analysis.

### Cell interaction evaluation

The possible interaction between cell populations was evaluated using CellphoneDB [56], based on the curated known ligand-receptor pairs implemented in the package. In brief, for every two given clusters, the genes encoding certain receptors or ligands which were expressed in more than 30% of cells in a specific cluster were chosen for downstream analysis, and the significance of a ligand-receptor pair between two clusters was calculated through the permutation test by randomly assigning the cluster labels of each cell 1,000 times. An empirical P value was determined by the rank of the actual average expression of a given ligand and receptor pair in two clusters among the 1,000 permutated results.

### Pseudo-time trajectory construction

Pseudo-time trajectory was constructed using Monocle v2.6.4 R package [57, 58]. The top 50 differentially expressed genes in each cluster identified using *FindAllMarkers* in Seurat were combined and used for dimension reduction (DDRTRee) and cell ordering.

### Statistical analysis and data availability

Statistical analyses were performed in R 3.4.3 (foundation for statistical computing, or functions implemented in Seurat). Detailed descriptions are specified in the text.

### Validation in independent datasets of 276 CML patients

Gene expression profiles of CML patients (n = 59) who had received imatinib treatment were obtained from the GEO database (GSE14671) [28]. Forty-one were imatinib nonresponders (NR) while 18 were responders (R). Samples of each CML were collected before the initiation of imatinib therapy. Gene expression profiles were obtained with the Affymetrix Human Genome U133 Plus 2.0 Array. The robust multiarray averaging method (RMA) was used to analyze the RNA expression microarray of each CML sample. The expression levels of each gene were log2 transformed. The correlation coefficients between the gene expression profiles of the 59 CML patients and the Clu-CD34 (or Clu-MPO) gene expression signatures (Supplementary Table 4) were calculated using the Pearson correlation. The correlation coefficients of the 41 imatinib nonresponders and the 18 responders were further used to build ROC curves with the pROC package (R 3.4.3).

Similarly, 3 other independent datasets with a total of 217 CML patients were analyzed to achieve significant scientific results. The clinical characteristics of the cohorts are the following: 1) imatinib therapy (GSE130404, 96 CML patients) [29]: 13 imatinib nonresponders and 83 responders; 2) dasatinib therapy (GSE33224, 14 CML patients) [30]: 8 dasatinib nonresponders and 6 responders; and 3) different phases of CML (GSE4170, 107 CML patients) [31]: 17 accelerated phase (AP), 33 (blast crisis (BC) and 57 chronic phase (CP) CML patients. The first two cohorts were used to build receiver operating curves (ROC).

## Supplementary Material

Supplementary Figures

Supplementary Table 1

Supplementary Table 2

Supplementary Table 3

Supplementary Table 4
