# Supplementary material for: Single cell sequencing reveals cell populations that predict primary resistance to imatinib in chronic myeloid leukemia
Source: Aging (Albany NY). 2020 Nov 23;12(24):25337–55. doi: 10.18632/aging.104136 (PMC7803567; doi:10.18632/aging.104136)
Supplement: Supplementary Figures [file aging-12-104136-s001.pdf]

SUPPLEMENTARY FIGURES

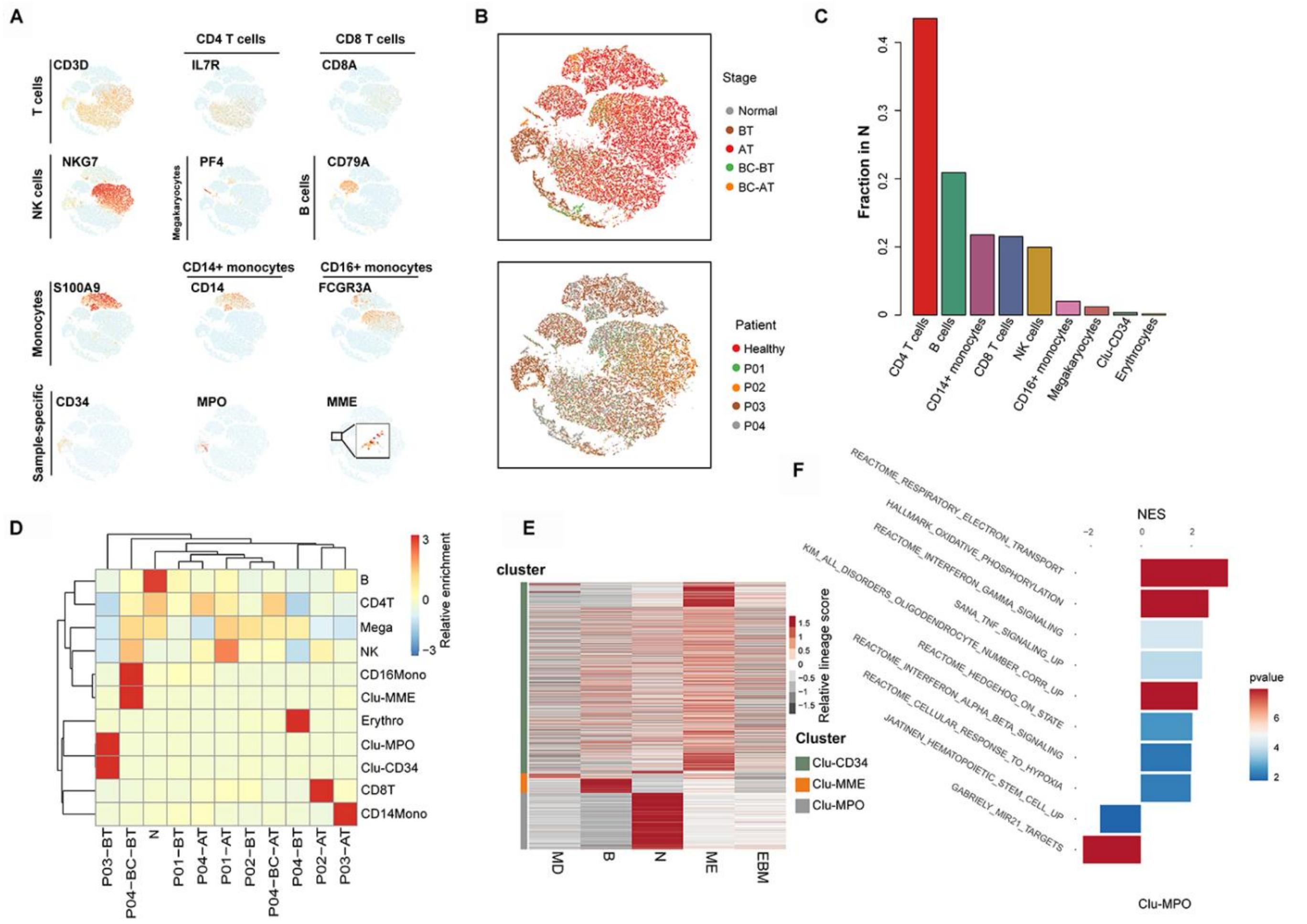

Supplementary Figure 1. (**A**) TSNE plots showing the cell type specific markers in the sequenced dataset. (**B**) TSNE plots of the sequenced dataset colored by sample types (top) and clinical stages (bottom). (**C**) Bar plots showing the fraction of different lineages from the healthy donor sample. The number of cells in each lineage is indicated. (**D**) Heatmap showing enrichment of samples in each cluster. Enrichment scores were calculated using the Fisher's exact test and indicated by log10(Odd Ratio). (**E**) Heatmap showing the lineage potential scores of each stem/progenitor cell towards the different development directions (adopted from Velten et al. [12] as shown in Supplementary Table 3) EBM: esophils/basophils/mast cell; N: neutrophils; ME: megakyrocytes/erythrocytes. B: B cells, MD: multiple direction. (**F**) Bar plots displaying the GSEA result on the ordered expression profile in Clu-MPO. X-axis indicates the normalized enrichment score (NES) and colors indicate the -log10(P value).

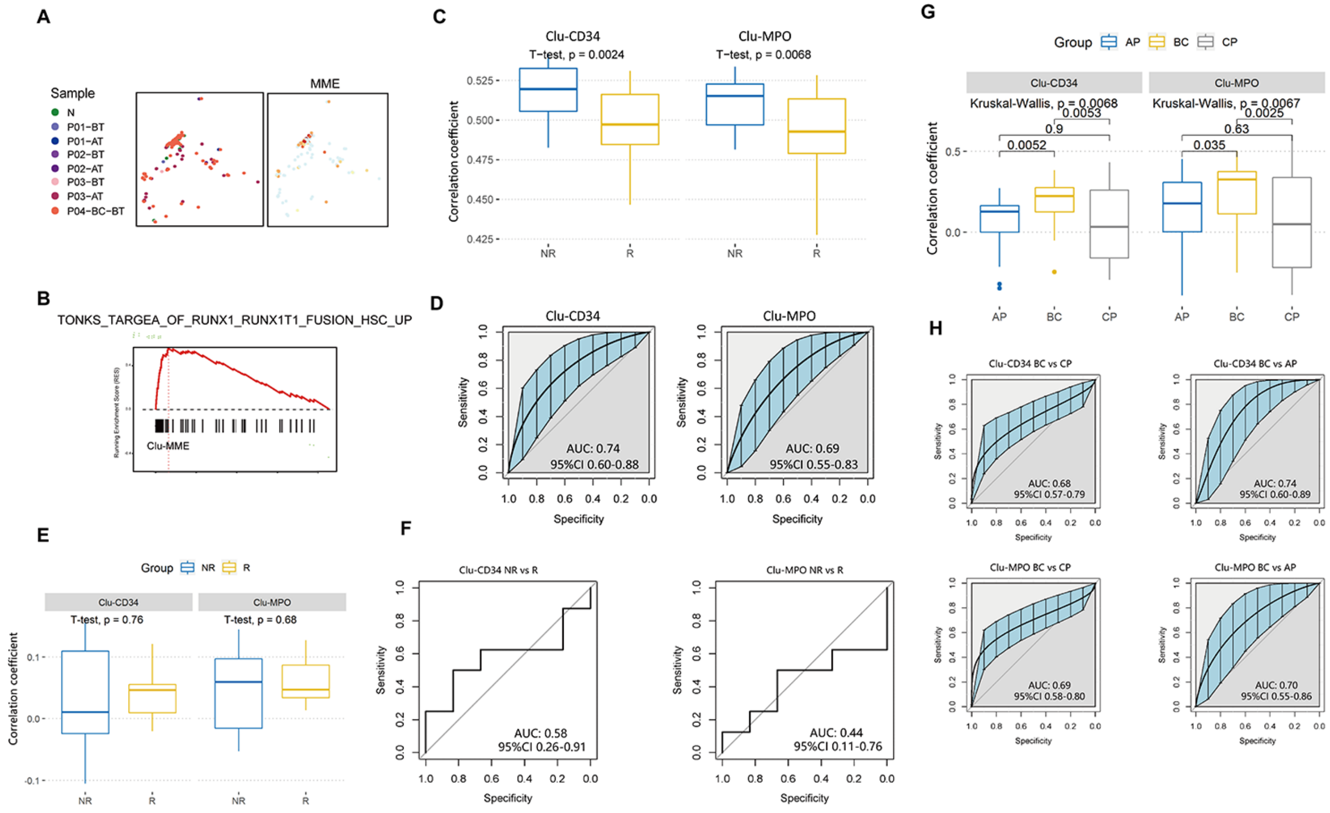

Supplementary Figure 2. (**A**) TSNE plots showing the sample origins (left) and expression of MME of cells in the Clu-MME cluster. (**B**) Gene set enrichment analysis revealing an enriched signature of RUNX1-RUNX11 fusion in Clu-MME. (**C**) Box plots comparing the correlation coefficients of Clu-CD34 (left) and Clu-MPO (right) between 41 imatinib nonresponders (NR) and 18 responders (R). The correlation coefficients were calculated using Pearson correlation of gene expression signatures of these two clusters with the gene expression profile for each CML patient treated with imatinib (see Methods). (**D**) ROC curves illustrating the classification performance of Clu-CD34 (left) and Clu-MPO (right) gene expression signatures of 41 imatinib nonresponders and 18 responders. The blue shade denotes the 95% confidence interval of the sensitivity at a given specificity point. AUC, area under the ROC curve are indicated. (**E**) Box plots comparing the correlation coefficients of Clu-CD34 (left) and Clu-MPO (right) between 8 dasatinib nonresponders (NR) and 6 responders (R) from GSE33224. The correlation coefficients were calculated using Pearson correlation of gene expression signatures of these two clusters with the gene expression profile in each CML patient treated with imatinib (see Methods). (**F**) ROC curves illustrating the classification performance of Clu-CD34 (left) and Clu-MPO (right) gene expression signatures of 8 dasatinib nonresponders (NR) and 6 responders (R) from GSE33224. The blue shade denotes the 95% confidence interval of the sensitivity at a given specificity point. AUC, area under the ROC curve are indicated. (**G**) Box plots comparing the correlation coefficients of Clu-CD34 (left) and Clu-MPO (right) for 17 AP (accelerated phase) CML patients, 33 BC (blast crisis) CML patients and 57 CP (chronic phase) CML patients from GSE4170. The correlation coefficients were calculated by Pearson correlation of gene expression signature of these two clusters with the gene expression profile in each CML patient treated with imatinib (see Methods). (**H**) ROC curves illustrating the classification performance of Clu-CD34 (up) and Clu-MPO (down) gene expression signatures for 17 AP (accelerated phase), 33 BC (blast crisis) and 57 CP (chronic phase) CML patients from GSE4170. The blue shade denotes the 95% confidence interval of the sensitivity at a given specificity point. AUC, area under the ROC curve are indicated.

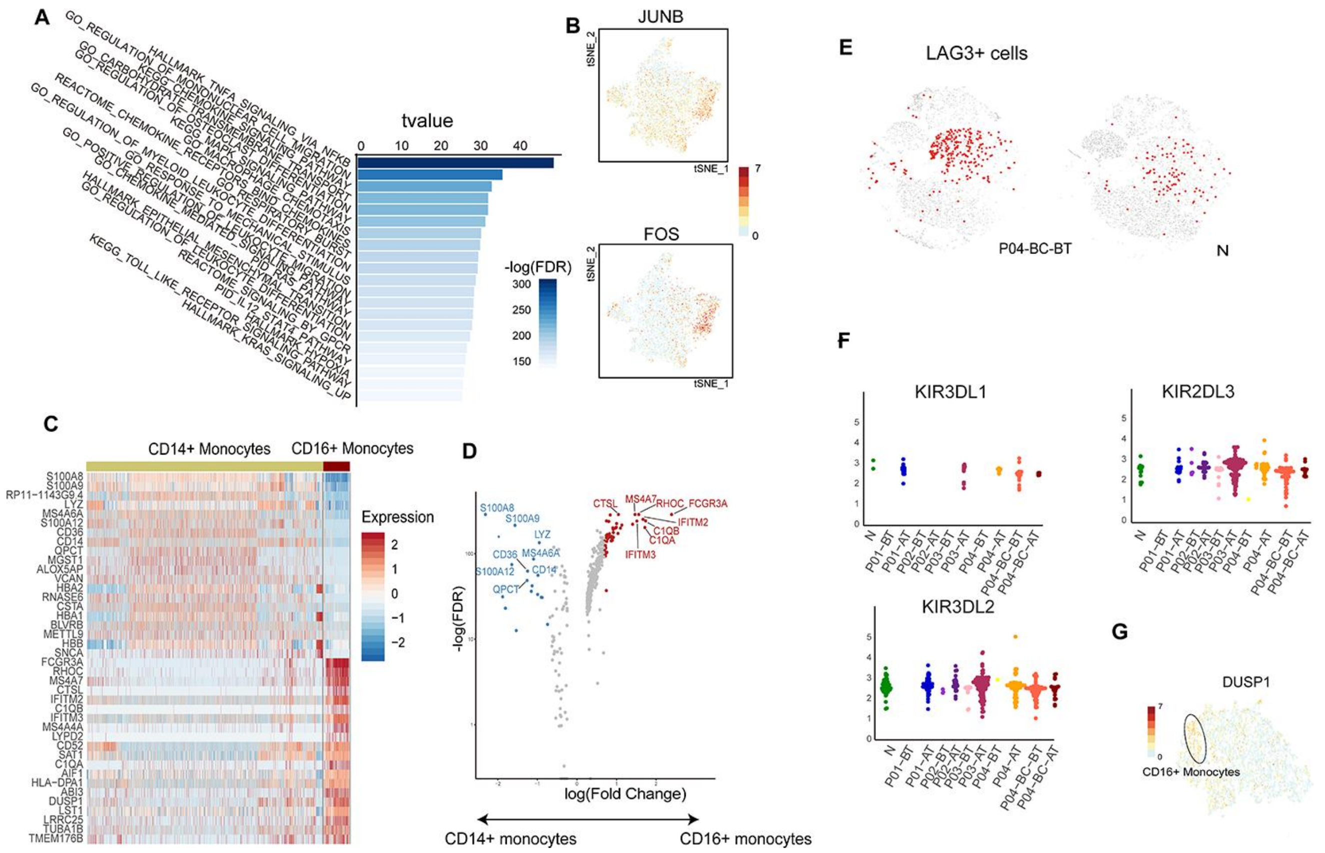

Supplementary Figure 3. (**A**) Bar plots displaying the GSVA *t* values of top ranked gene sets. Bars are colored by the FDR *Q* value. (**B**) TSNE plots showing expression of JUNB and FOS in CD14+ monocytes. (**C**) Heatmap comparing the expression profiles for all CD14+ and CD16+ monocytes. (**D**) Scatter plot showing the highly-expressed markers in CD14+ monocytes and CD16+ monocytes, separately. Significant markers (FDR<0.05, fold change > 2) are colored as red in CD16+ monocytes and blue in CD14+ monocytes. (**E**) TSNE plots displaying LAG3 expression status of T cells/NK cells in P04-BC-AT in comparison with N. Cells positive for LAG3 were indicated as red. (**F**) Beeswarm plots showing the expression of other KIRs. The number of cells in each sample is indicated. (**G**) TSNE plot showing the enrichment of DUSP1 expression in CD16+ monocytes among all monocytes. Dashed line marks the cluster of CD16+ monocytes.
