# Supplementary material for: Single cell sequencing reveals cell populations that predict primary resistance to imatinib in chronic myeloid leukemia
Source: Aging (Albany NY). 2020 Nov 23;12(24):25337–55. doi: 10.18632/aging.104136 (PMC7803567; doi:10.18632/aging.104136)
Supplement: Supplementary Table 2 [file aging-12-104136-s003.pdf]

SUPPLEMENTARY TABLE

Supplementary Table 2. Number of cells in each cluster across samples.

	N	P01-AT	P01-BT	P02-AT	P02-BT	P03-AT	P03-BT	P04-AT	P04-BC-AT	P04-BC-BT	P04-BT	Total
B	1045	526	54	197	44	872	99	237	103	377	24	3578
CD4T	2176	2100	171	1309	354	2191	120	1629	759	1535	4	12348
CD8T	576	1027	31	2674	243	1694	67	591	48	167	1	7119
CD14Mono	589	481	84	102	59	3776	189	619	186	434	49	6568
CD16Mono	101	33	17	13	7	48	2	80	6	429	4	740
Erythro	9	3	91	2	256	55	425	30	374	285	698	2228
Mega	61	75	3	17	17	19	5	5	20	58	9	289
Clu-MME	8	2	1	3	1	31	3	0	0	135	0	184
NK	497	1517	53	864	178	1236	105	606	248	1040	9	6353
Clu-MPO	0	0	5	0	28	29	451	0	0	5	9	527
Clu-CD34	20	12	52	5	141	101	1375	4	5	50	24	1789
**Total**	5082	5776	562	5186	1328	10052	2841	3801	1749	4515	831	**41723**
